# Independent effect of postoperative neutrophil-to-lymphocyte ratio on the survival of pancreatic ductal adenocarcinoma with open distal pancreatosplenectomy and its nomogram-based prediction

**DOI:** 10.7150/jca.35856

**Published:** 2019-10-15

**Authors:** Ning Pu, Hanlin Yin, Guochao Zhao, Abulimiti Nuerxiati, Dansong Wang, Xuefeng Xu, Tiantao Kuang, Dayong Jin, Wenhui Lou, Wenchuan Wu

**Affiliations:** Department of General Surgery, Zhongshan Hospital, Institute of General Surgery and Shanghai Medical College, Fudan University, Shanghai, 200032, People's Republic of China

**Keywords:** PDAC, ODPS, neutrophil-to-lymphocyte ratio, prognosis, nomogram

## Abstract

**Background:** The prognosis of pancreatic ductal adenocarcinoma (PDAC) remains poor. Open distal pancreatosplenectomy (ODPS) is prevalent in the patients of early PDAC located in pancreatic body or tail. However, the models for relapse or survival prediction in those patients are still limited. Postoperative neutrophil-to-lymphocyte rate (poNLR), a novel inflammation-based score, has been formulated to analyze the prognostic significance in PDAC patients with ODPS. Therefore, this study aims to generate a valuable prognostic nomogram for PDAC following ODPS.

**Methods:** We retrospectively enrolled 97 patients of PDAC undergoing ODPS in this study. The Cox proportional hazards regression methodology was used in univariate and multivariate survival analyses to identify significant independent prognostic factors. The prognostic nomograms integrating poNLR into the American Joint Commission on Cancer (AJCC) staging system (8th edition) for predicting overall survival (OS) and relapse free survival (RFS) were established to achieve superior discriminatory abilities. Further, these prognostic nomograms were verified according to concordance index (C-index), calibrations and decision curve analyses (DCA).

**Results:** The optimal cut-off value of poNLR for assessing OS determined by X-tile program was 14.1. Higher poNLR was associated with higher postoperative neutrophil (poNeutrophil), lower postoperative lymphocyte (poLymphocyte), lower preoperative lymphocyte-to-monocyte rate (preLMR) and higher △NLR (postoperative-preoperative NLR). In the univariate and multivariate analysis, poNLR was identified as an independent prognostic indicator for OS and RFS (P=0.044 and 0.028, respectively) and patients with higher poNLR level were probable to have shorter OS and RFS. Compared with the TNM staging system of the AJCC 8th edition, the nomogram comprising of poNLR and AJCC 8th edition exhibited superior predictive accuracy for OS and RFS.

**Conclusions:** poNLR can be a proven, inexpensive and novel survival predictor of PDAC patients with ODPS. One more advanced and accurate predictive model will be achieved to assist in risk stratification via the incorporation of poNLR into nomograms.

## Introduction

Open distal pancreatosplenectomy (ODPS) is the standard surgical operation for pancreatic ductal adenocarcinoma (PDAC) which located in pancreatic body or tail. Despite aggressive surgical therapy, PDAC still causes the fourth leading cancer-related deaths^1^, ^2^. In PDAC patients with ODPS, suitable systemic stratification and patient enrollment for treatment remain challenges. Considering this, one more novel prognostic biomarkers are needed urgently to assist in the decision-making of treatment and surveillance to obtain uppermost improvements for PDAC patients.

Some evidences have verified the value of systematic inflammation response in predicting the progression of malignant cancers and survival of patients, which has received considerable interest owing to its simplicity, convenience and inexpensiveness^3^. Numerous studies have showed that the increased preoperative neutrophil-to-lymphocyte ratio (preNLR) owns a dominant prognostic value in several diseases, such as gastric, colorectal, hepatocellular, pancreatic, lung cancer and so on^4^-^9^ . In addition, amounts of studies have showed the prognostic value of preNLR in localized or advanced pancreatic cancer, and an elevated preNLR was significantly correlated to shorter survival or PDAC occurrence^4^, ^10^-^12^. The cut-off value of preNLR as previously literatures reported was around 2.0-5.0^4^, ^13^, ^14^. However, few studies reported prognostic value of elevated NLR in ODPS patients. In particular, most studies focused on preNLR as a predictor of survival in PDAC, while no study assessed the correlation between postoperative neutrophil-to-lymphocyte ratio (poNLR) and prognosis after ODPS for PDAC. The poNLR may better reflect the prognosis-related systemic immune response and the stress suffering from surgery according to several recent reports^15^-^18^.

Taken together, we logically hypothesized that a novel inflammatory biomarker, poNLR, can predict the survival outcome after ODPS for PDAC. We dug into the connections between prognostic significance of poNLR and clinicopathological characteristics in PDAC undergoing ODPS to evaluate its values in prediction of overall survival (OS) and relapse free survival (RFS). Additionally, an innovative prognostic nomogram model incorporating poNLR and AJCC 8th staging system was formulated to predict the prognosis.

## Patients and Methods

### Patient data

We retrospectively collected and analyzed 97 PDAC patients from March 2012 to March 2016 who underwent ODPS at Zhongshan Hospital, Fudan University. All the patients had received the optimal chemotherapy was provided by multiple disciplinary teams by synthesizing the patients' tumor burden, physical and financial condition; The inclusion and exclusion criteria were as follows: (1) patients who underwent ODPS and confirmed as PDAC by exactly pathological diagnosis, and without unknown origins or distant metastasis;(2) no preoperative anti-inflammatory or anti-cancer treatments; (3) no concurrence or history of other malignant cancers; (4) no evidence of preoperative infection, hematological or inflammatory diseases; (5) sufficient clinicopathological and follow-up data. The protocol of this study was approved by the Clinical Research Ethic Committee of Zhongshan Hospital, Fudan University and all informed consents were obtained.

Conventional clinicopathological parameters were examined within preoperative 3 days, while blood routine test and biochemical test were also examined within postoperative one day routinely, such as preoperative CA19-9 (preCA19-9), CEA (preCEA), monocyte (preMonocyte) and preoperative or postoperative neutrophil (preNeutrophil or poNeutrophil), lymphocyte (preLymphocyte or poLymphocyte), albumin (preALB or poALB), alanine aminotransferase (preALT or poALT), gamma-glutamyltransferase (preGGT or poGGT) and lactate dehydrogenase (preLDH or poLDH). The clinical tumor stage for each patient was evaluated by postoperative histopathological examination and clinical assessment in accordance with the AJCC 8th staging system. Data were obtained from hospital record system. The NLR was calculated by dividing the blood neutrophil counts by the blood lymphocyte counts and △NLR was calculated by subtracting preNLR from poNLR.

### Follow-up

Postoperative follow-up schedules were carried out with all patients according to the NCCN Clinical Practice Guidelines, including physical, laboratory and radiological examinations. In detail, blood routine and biochemical tests, and tumor biomarkers were examined quarterly within first 2 years, biannually within next 3 years, and yearly thereafter. Patients routinely underwent enhanced abdominal computed tomography (CT) scans or magnetic resonance imaging (MRI) in every 6 months. In addition, all patients were followed up via telephone call and the last follow-up work was finished on December 30, 2016. The OS was defined as the period between surgery and December 2016 or death and the date of surgery to tumor relapse or December 2016 was considered as RFS.

### Statistical procedures

SPSS 23.0 statistical package (SPSS Inc., Chicago, IL, USA) and R 3.4.4 project were used for analysis (Bell Laboratories, Murray Hill, NJ, USA). The optimal cut-off value for poNLR was calculated by X-tile program (Yale University, New Haven, CT, USA). The correlations between poNLR and clinicopathologic variables were analyzed using Pearson's χ2 test or Fisher's exact test as appropriate. Log-rank test and Kaplan-Meier method were used to analyze and depict survival curves. Independent prognostic factors were identified by using Cox proportional hazards regression model for multivariate analysis, and significant difference was found when p value was less than 0.05.

Novel prognostic nomograms for OS and RFS based on poNLR and AJCC 8th staging system were respectively established. Concordance index(C-index), calibration curve and decision curve analysis (DCA) were further used to evaluated predictive performance as previously described^3^, ^19^, ^20^.

## Results

### Clinicopathological characteristics

According to whole criteria above, 97 patients with ODPS were finally collected. The detailed clinical and pathological characteristics were displayed in Table 1. Fifty-two males and forty-five females consisted of the gender distribution. The population's median age was 64 (ranging between 35 and 81) years. The median OS and RFS was 17 months (ranges, 6-78 months) and 10 months (ranges, 1-72 months), respectively. Furthermore, 1-, 2- and 3-year OS rates were 68.4%, 30.7% and 14.8% respectively, while 1- and 2-year RFS rates were 37.1% and 14.0% respectively. Among these patients, 29 patients suffered from well or moderate pathological differentiation, whereas 68 patients were observed in poor differentiation. From the AJCC 8th staging system, number of patients of I, II A, II B and III stage was 24, 31, 25 and 17 respectively.

### Identification of the optimal cut-off value for poNLR

**Figure 1:**
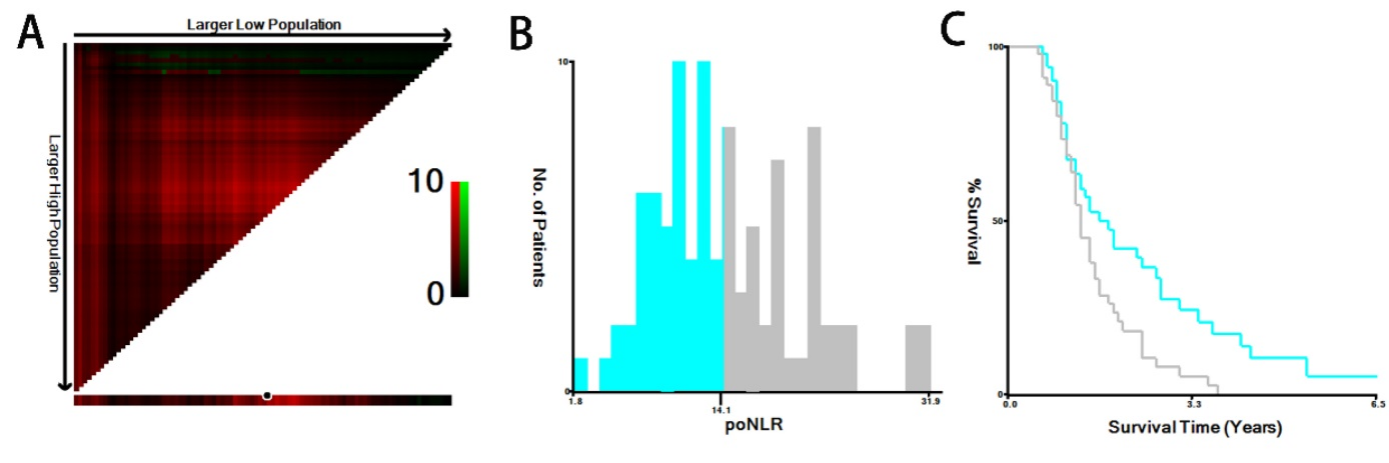
** X-tile analysis of OS.** The optimal cut-off value with dichotomies for poNLR was 14.1 (A, B, C). OS, overall survival; poNLR, postoperative neutrophil-to-lymphocyte rate.

The median poNLR of the collected population was 13.8 (ranges: 1.8-31.9). Using X-tile program, a biostatistical tool, we further determined 14.1 as an optimal cut-off value of poNLR for survival analysis (Figure 1), as well as other inflammation-based scores displayed in Table 1, such as albumin-to-gamma glutamyltransferase ratio (AGR), lymphocyte-to-monocyte ratio (LMR), albumin-to-lactate dehydrogenase ratio (ALR) and △NLR. Thus, we divided the cohort into two groups in line accordance with the poNLR (poNLR>14.1 as a high-risk group and poNLR≤14.1 as a low-risk group).

### Correlation between poNLR and clinicopathologic characteristics in PDAC with ODPS

As shown in Table 2, clinicopathological characteristics were compared between patients in high-poNLR group and low-poNLR group. Significant positive associations were found between high-poNLR group and elevated poNeutrophil, declined poLymphocyte or higher △NLR (p<0.001). Intriguingly, high-poNLR group was also associated with lower preLMR (p=0.025). However, no significant differences were found between two cohorts with other clinicopathological or inflammatory parameters.

### Association of poNLR with OS and RFS for PDAC with ODPS

**Figure 2:**
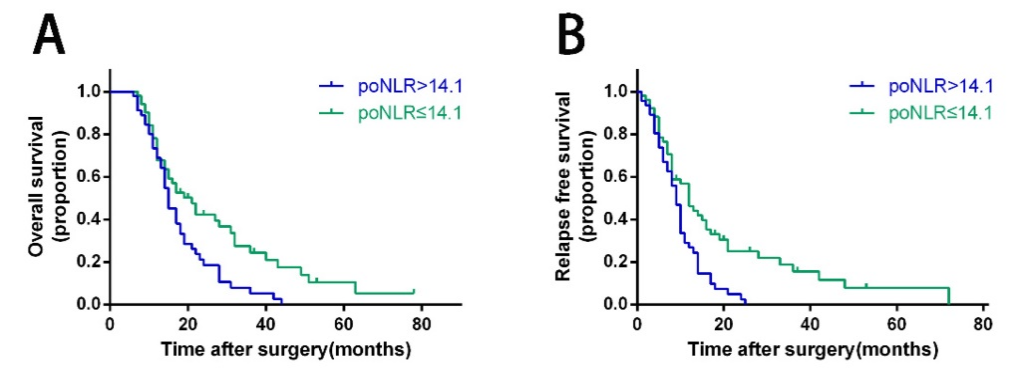
** Kaplan-Meier survival curves for OS and RFS of PDAC patients with ODPS according to poNLR levels.** Patients of PDAC with poNLR more than 14.1 were inclined to significantly poorer OS (A) and RFS (B). The *P*-values were analyzed by the log-rank test. OS, overall survival; RFS, relapse free survival; PDAC, pancreatic ductal adenocarcinoma; ODPS, open distal pancreatosplenectomy; poNLR, postoperative neutrophil-to-lymphocyte rate.

In PDAC patients with ODPS, Kaplan-Meier curves revealed poNLR larger than 14.1 was correlated with poor prognosis significantly than low-poNLR group in terms of OS and RFS (Figure 2, p=0.008 and 0.002 respectively). In high-risk group, OS rates at 1, 2 and 3 years were 68.9%, 18.5%, 5.3%, while 67.8%, 42.3%, 24.4% in low-risk group. In addition, RFS rates at 1 and 2 years were 26.8%, 2.4% in high-risk group compared to 46.3%, 23.4% in low-risk group.

The univariate analysis showed that advanced TNM stage (p <0.003), decreased poALP (p=0.035), preLMR (P=0.013) and elevated preMonocyte (p=0.019), poNLR (p=0.012), were considered as significant risk factors in OS, as well as preNeutrophil (p=0.049), poNeutrophil (p=0.029) and △NLR (P=0.026) in RFS analysis (Table 1). TNM stage (p=0.001, hazard ratio [HR]=2.177; 95% confidential interval [CI]:1.380-3.435 and p=0.001, HR=2.191; 95%CI:1.396-3.439 respectively), and poNLR level (p=0.044, HR=1.618; 95%CI:1.014-2.582 and p=0.028, HR=3.278; 95%CI:1.141-9.999 respectively) remained as independent prognostic indicators in multivariable analysis. Moreover, preMonocyte counts (p=0.027, HR=2.214; 95%CI:1.093-4.486) was also confirmed for OS (Table 1).

### Prognostic nomograms incorporating poNLR and AJCC 8th staging system

**Figure 3:**
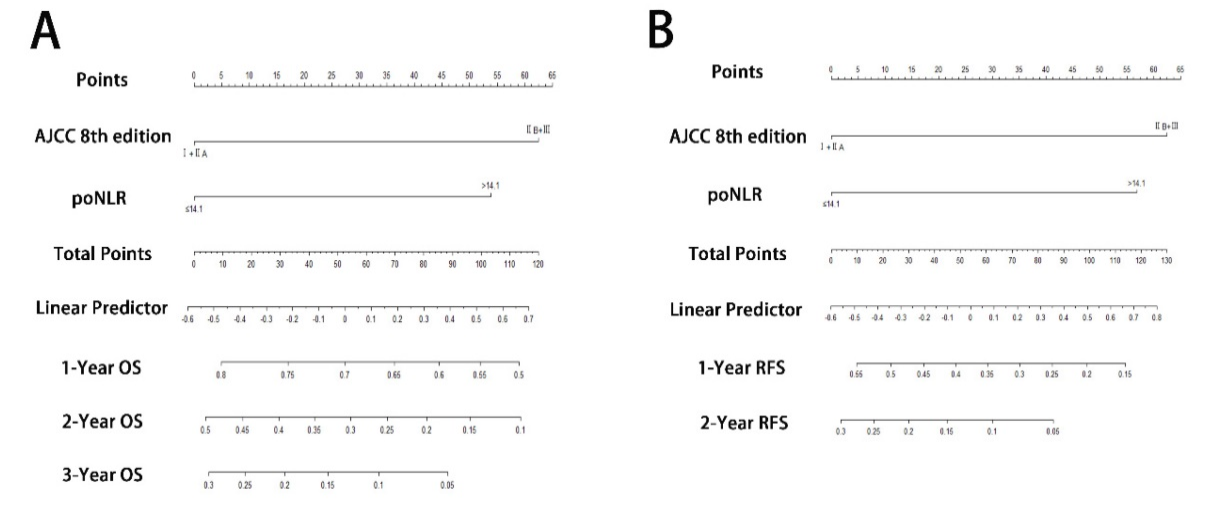
** Prognostic nomograms for PDAC with ODPS.** The nomograms predicted OS (A) and RFS (B) in patients of PDAC undergoing ODPS. PDAC, pancreatic ductal adenocarcinoma; ODPS, open distal pancreatosplenectomy; OS, overall survival; RFS, relapse free survival; poNLR, postoperative neutrophil-to-lymphocyte rate; AJCC, American Joint Committee on Cancer.

To predict OS and RFS, two nomograms, one more accurate predictive model, were created by integrating two independent prognostic indicators, poNLR and AJCC 8th staging system, according to multivariate Cox regression models (Figure 3A and 3B). C-indices for OS and RFS prediction with the formulated nomograms were 0.658 (95% CI, 0.643-0.673) and 0.644 (95% CI, 0.629-0.659) respectively, as well as C-indices for other variables shown in Table 3, which indicated that the nomograms incorporating poNLR and AJCC 8th staging system had higher C-indices and better predictive ability for OS and RFS than that of AJCC 8th staging system alone.

**Figure 4:**
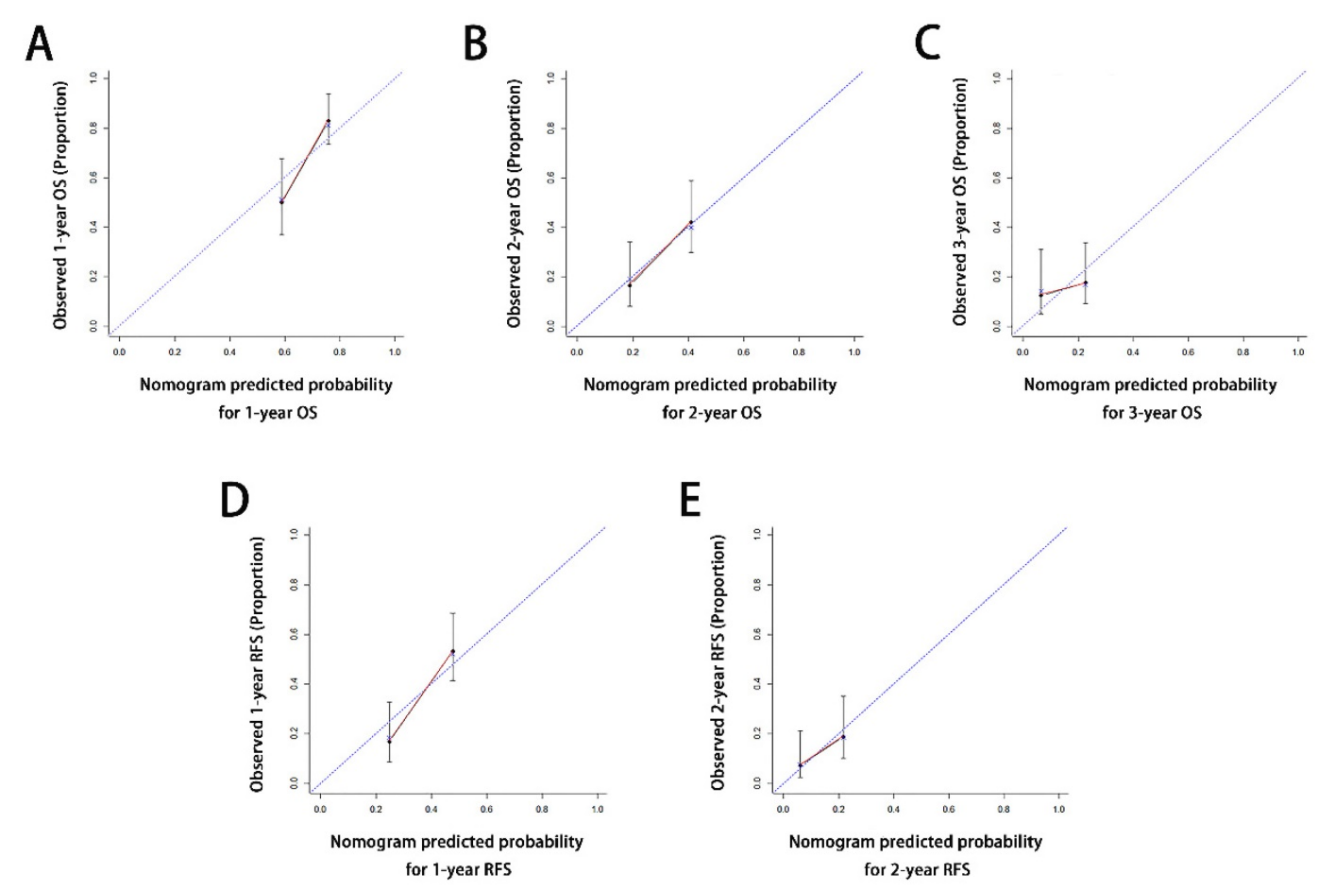
** Calibration curves for the formulated nomograms.** The calibration curves predicted OS at 1 years (A), 2 years (B) and 3 years (C) and RFS at 1 years (D), 2 years (E) in the whole cohort. Nomogram-predicted probability of OS or RFS was plotted on the x axis and the observed OS or RFS was plotted on the y axis. OS, overall survival; RFS, relapse free survival.

Calibration curves for probability of OS at 1, 2, 3 years (Figure 4A-4C) and RFS at 1, 2 years (Figure 4D-4E) after ODPS showed excellent consistency between nomogram-prediction and actual observation.

**Figure 5:**
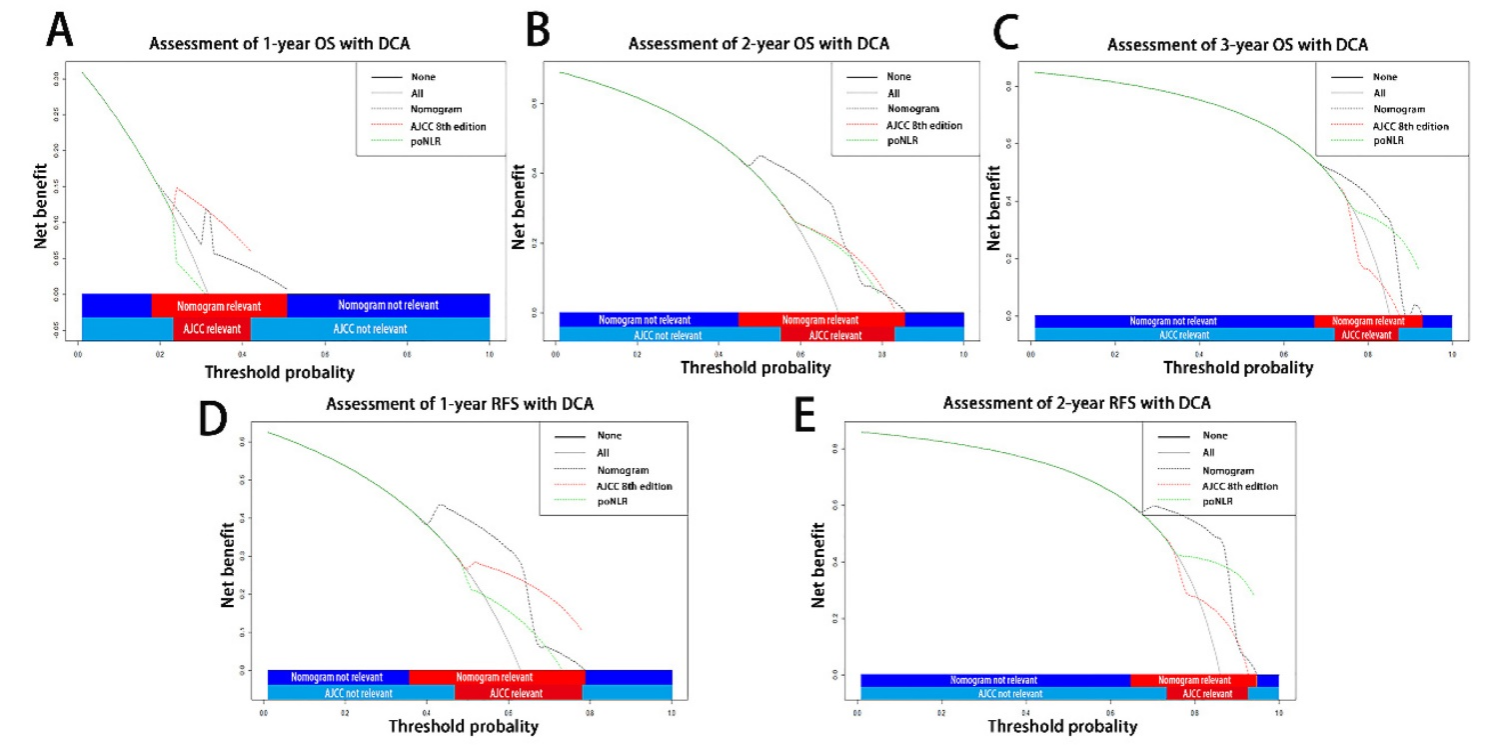
** Decision curve analyses for different predictive models.** Decision curve analyses depicted the clinical net benefit in pairwise comparisons across the different models. Nomograms were compared to the AJCC 8th edition in terms of 1-(A), 2-(B), 3-(C) year OS and 1-(D), 2-year(E) RFS in the enrolled cohorts. The dashed lines indicated the net benefit of the predictive models across a range of threshold probabilities (black: nomograms; red: AJCC TNM staging system of 8th edition; green: poNLR). The horizontal solid black line represented the assumptions that no patient would experience the event, and the solid grey line represented the assumption that all patients would experience the event. On decision curve analyses, the nomograms for OS and RFS revealed better net benefits compared with AJCC 8th edition across a wider range of threshold probabilities. OS, overall survival; RFS, relapse free survival; poNLR, postoperative neutrophil-to-lymphocyte rate; TNM, tumor node metastasis; AJCC, American Joint Committee on Cancer

On decision curve analysis (DCA) as our previously applied^3^, ^19^, ^20^, the nomogram incorporating poNLR and AJCC 8th staging system yielded wider threshold probability and better net benefit than AJCC 8th staging system alone, which indicated higher predictive power for OS and RFS prediction (Table 3; Figure 5A-5E). Meanwhile, higher threshold probability represented better estimation for decision outcomes.

## Discussion

Among this study, patients of PDAC operated with ODPS were analyzed. To our knowledge, we formulated poNLR, a convenient and original inflammation-based score constituting neutrophil and lymphocyte counts, as a precise predictor in PDAC patients undergoing ODPS. In analysis with the clinicopathological characteristics, patients with higher poNLR (>14.1) had higher poNeutrophil counts as well as lower poLymphocyte counts. In addition, they also had correlations with △NLR and preLMR level. Next, significant associations were found between poNLR and OS or RFS according to univariate and multivariate analysis. Furthermore, nomograms containing poNLR and AJCC 8th staging system validated by calibrations and DCA manifested improved prediction power in contrast to AJCC 8th staging system alone.

The aforementioned results imply that the risk of cancer-related relapse or death increases after ODPS with the elevation in postoperative day one NLR. It is gradually accepted that there is a very strong link between systemic inflammation and development of PDAC^21^. The blood parameters reflect the anti-factor and promoting-factor of inflammatory response and immune response to the cancer^16^. Our previous study had investigated the changes of microenvironment in PDAC, which showed the prognosis-related changes of regulatory T cells and CD8^+^ T cells^22^. Our present research indicated that elevated poNLR was an independent predictive factor to the prognosis of PDAC undergoing ODPS. Balkwill F et al.^23^ believed that inflammation could drive cancer progression rather than suppress the tumorigenesis or metastasis. The active inflammation concluded as a bad prognosis to patients with cancers. It was proposed that tumor cells stimulated neutrophils by secreting interleukin-8, which dissolved extracellular matrix to help tumor cells traverse across the vasculature and spread to a wider space^24^. Besides that, neutrophils could promote tumor growth by inflammatory markers like vascular endothelial growth factor (VEGF), proteases, NF-kB^25^. Just as the membrane transports of serum electrolytes, when the serum concentration reached to an aberrant level, the intrinsic balance would disrupt. So postoperative day one higher neutrophil counts may immediately change the latent microenvironment of cancer, which contributed to the recurrence and progression^26^.

In ODPS procedure, splenectomy and the surgical trauma contributing to the alternation of peripheral blood parameters should be taken into consideration. Exstirpation of the spleen makes unignorable changes in immune status. The neutrophils' proportion and absolute counts increase, whereas the lymphocytes' proportion and absolute counts are not significantly changing or even decrease^27^, ^28^. All these may lead to an increasing ratio of the poNLR. Simultaneously, we determined the optimal cut-off value of poNLR as 14.1. Although Stotz M et al.^29^ reported that preNLR≥5 was selected as threshold for distinguish the high/low-risk group in pancreatic cancer, our study failed in obtaining the optimal cut-off value of preNLR and △NLR could not be an independent predictor through univariate and multivariate analysis. In addition, comparing with preNLR which may indicate tumor progress, the poNLR was appropriate to provide more precise systemic condition of post-operation like connective tissue disorders, frequently physical examination or mental stress^16^. We also found that preLMR had a significantly correlation with poNLR, which was consistent with the previous studies that verified high preMonocyte counts and low preLMR level could predict a poor prognosis in PDAC^30^. Combining with our study, we speculated that there were potential relationships between monocytes and neutrophils in the PDAC microenvironment^31^.

Although a series of inflammation-based scores appeared to be prognostic factors in diverse malignances^3^, ^32^, inflammation factors were failed to be incorporated into AJCC 8th staging system or other existed predictive model in PDAC. Intriguingly, AJCC 8th staging system integrating poNLR in Table 3 indicated that the incorporated nomograms led to higher C-indices than that of AJCC 8th staging system alone. This meant that our findings may provide a novel clue to predict the prognosis of PDAC with ODPS more accurately.

There were still several limitations in our study. Firstly, this remained as a single-center retrospective study essentially, potential bails must be considered, and a multicenter and large-scale validation group was needed. Secondly, patients undergoing ODPS procedure may have differential surgical trauma because of different physical conditions and surgeons' operations.

In conclusion, this study verified the links between high poNLR and poor prognosis. The poNLR, deriving from inexpensive and easy-accessible clinical routine blood tests, could be a quite effective predictor for PDAC with ODPS. Moreover, the established nomograms integrating poNLR and AJCC 8th staging system provided higher predictive accuracy. However, because of the limitations, further studies were warranted to authenticate our findings.

## Figures and Tables

**Table 1:** Clinicopathological characteristics of patients with PDAC: univariate and multivariate analysis.

Variables	Patients (n=97)	OS			RFS		
		Univariate *P*-value	Multivariate *P*-value	Multivariate HR (95% CI)	Univariate *P*-value	Multivariate *P*-value	Multivariate HR (95% CI)
Gender, male/female	52/45	0.676	NA		0.430	NA	
Age, years(medium,range)	64, 35-81	0.309	NA		0.317	NA	
Differentiation, well and moderate/poor	29/68	0.221	NA		0.608	NA	
Vascular invasion, yes/no	15/82	0.766	NA		0.710	NA	
Tumor size, ≤ 4/ > 4 cm	42/55	0.515	NA		0.133	NA	
TNM stage, I+IIA/IIB+III	55/42	**0.003**	**0.001**	2.177(1.380-3.435)	**0.001**	**0.001**	2.191(1.396-3.439)
preCA19-9, < 37/ ≥ 37 U/L	23/74	0.208	NA		0.307	NA	
preCEA, < 5/ ≥ 5 ng/mL	76/21	0.271	NA		0.602	NA	
preNeutrophil, ≤ 6.3/ >6.3 *10^9^/L	90/7	0.084	NA		**0.049**	0.346	1.506(0.643-3.532)
preLymphocyte, < 1.1/ ≥ 1.1 *10^9^/L	9/88	0.825	NA		0.959	NA	
preMonocyte, ≤ 0.6/ >0.6 *10^9^/L	87/10	**0.019**	**0.027**	2.214(1.093-4.486)	0.051	NA	
preALB, <35/ ≥ 35 g/L	3/94	0.811	NA		0.983	NA	
preALT, ≤ 50/ > 50 U/L	95/2	0.505	NA		0.836	NA	
preGGT, ≤ 60/ > 60 U/L	86/11	0.889	NA		0.606	NA	
preLDH, ≤245/ >245 U/L	93/4	0.440	NA		0.375	NA	
poNeutrophil, ≤ 11.65/ >11.65 *10^9^/L	28/69	0.075	NA		**0.029**	0.295	1.366(0.762-2.447)
poLymphocyte, < 1.1/ ≥ 1.1 *10^9^/L	51/46	0.449	NA		0.403	NA	
poALB, <35/ ≥ 35 g/L	45/52	0.195	NA		0.609	NA	
poALT, ≤ 50/ > 50 U/L	87/10	0.905	NA		0.882	NA	
poGGT, ≤ 60/ > 60 U/L	87/10	0.703	NA		0.830	NA	
poLDH, ≤245/ >245 U/L	50/47	0.886	NA		0.558	NA	
preLMR, ≤ 5.8/ >5.8	65/32	**0.013**	0.078	0.630(0.377-1.053)	**0.036**	0.150	0.704(0.436-1.135)
preAGR, ≤ 1.66/ >1.66	41/56	0.978	NA		0.432	NA	
preALR, ≤ 0.227/ >0.227	39/58	0.375	NA		0.232	NA	
poNLR, ≤ 14.1/ >14.1	51/46	**0.012**	**0.044**	1.618(1.014-2.582)	**0.003**	**0.028**	3.378(1.141-9.999)
poAGR, ≤0.92/ > 0.92	22/75	0.682	NA		0.566	NA	
poALR, ≤ 0.151/ >0.151	54/43	**0.035**	0.108	0.683(0.428-1.087)	**0.042**	0.152	0.714(0.450-1.132)
△NLR, ≤ 12.1/ >12.1	53/44	0.053	NA		**0.026**	0.137	0.431(0.142-1.309)

OS, overall survival; RFS, relapse free survival; NLR, neutrophil-to-lymphocyte ratio; NA, not available. P values <0.05 are highlighted in bold font.

**Table 2:** Correlation between poNLR and clinicopathological variables of patients with PDAC.

Variables	poNLR≤ 14.1 (n=51)	poNLR> 14.1 (n=46)	*P*-value
Gender, male/female	30/21	22/24	0.278
Age, < 60/ ≥ 60	16/35	14/32	0.921
Differentiation, well and moderate/poor	13/38	16/30	0.318
Tumor size, ≤ 4/ > 4 cm	21/30	21/25	0.657
TNM stage, I+IIA/IIB+III	28/23	27/19	0.707
preCA19-9, < 37/ ≥ 37 U/L	14/37	9/37	0.362
preCEA, < 5/ ≥ 5 ng/mL	37/14	39/7	0.144
poNeutrophil, ≤ 11.65/ >11.65 *10^9^/L	23/28	5/41	**<0.001**
poLymphocyte, < 1.1/ ≥ 1.1 *10^9^/L	12/39	39/7	**<0.001**
poALB, <35/ ≥ 35 g/L	25/26	20/26	0.585
poALT, ≤ 50/ > 50 U/L	47/4	40/6	0.612
poGGT, ≤ 60/ > 60 U/L	46/5	41/5	1
poLDH, ≤245/ >245 U/L	29/22	21/25	0.270
preLMR, ≤ 5.8/ >5.8	29/22	36/10	**0.025**
preAGR, ≤ 1.66/ >1.66	22/29	19/27	0.855
preALR, ≤ 0.227/ >0.227	18/33	21/25	0.299
poAGR, ≤0.92/ > 0.92	12/39	10/36	0.833
poALR, ≤ 0.151/ >0.151	26/25	28/18	0.328
△NLR, ≤ 12.1/ >12.1	50/1	3/43	**<0.001**

NLR, neutrophil-to-lymphocyte ratio; preCA19-9, preoperative cancer antigen CA19-9; preCEA, preoperative carcino embryonie antigen; poNeutrophil, postoperative neutrophil; poLymphocyte, postoperative lymphocyte; poALB, postoperative albumin; poALT, postoperative alanine aminotransferase; poGGT, postoperative gamma-glutamyltransferase; poLDH, postoperative lactate dehydrogenase; LMR, lymphocyte-to-monocyte ratio; AGR, albumin to gamma-glutamyltransferase ratio; ALR, albumin to lactate dehydrogenase ratio. P values <0.05 are highlighted in bold font.

**Table 3:** Discriminatory capabilities of nomogram and independent prognostic factors in patients with PDAC: C-indices in OS and RFS prediction.

Variables	OS		RFS	
	C-index	95% CI	C-index	95% CI
Nomogram (AJCC 8th edition +poNLR)	0.658	0.643-0.673	0.644	0.629-0.659
AJCC 8th edition	0.619	0.607-0.631	0.601	0.589-0.613
poNLR	0.556	0.543-0.569	0.567	0.555-0.579

OS, overall survival; RFS, relapse free survival; c-index, concordance index; NLR, neutrophil-to-lymphocyte ratio.

## Abbreviations

AGR: albumin-to-gamma glutamyltransferase ratio
ALB: albumin
AJCC: American Joint Commission on Cancer
ALT: alanine aminotransferase
C-index: concordance index
CT: computed tomography
DCA: decision curve analysis
GGT: gamma-glutamyltransferase
LDH: lactate dehydrogenase
LMR: lymphocyte-to-monocyte ratio
MRI: magnetic resonance imaging
NLR: neutrophil-to-lymphocyte ratio
ODPS: open distal pancreatosplenectomy
OS: overall survival
PDAC: pancreatic ductal adenocarcinoma
RFS: relapse free survival
VEGF: vascular endothelial growth factor
